# Electrical Pumping of Perovskite Diodes: Toward Stimulated Emission

**DOI:** 10.1002/advs.202101663

**Published:** 2021-07-08

**Authors:** Changsoon Cho, Tobias Antrack, Martin Kroll, Qingzhi An, Toni R. Bärschneider, Axel Fischer, Stefan Meister, Yana Vaynzof, Karl Leo

**Affiliations:** ^1^ Dresden Integrated Center for Applied Physics and Photonic Materials (IAPP) Technische Universität Dresden Nöthnizer straße 61 Dresden 01187 Germany; ^2^ Centre for Advancing Electronics Dresden (cfaed) Technische Universität Dresden Helmholtzstraße 18 Dresden 01069 Germany; ^3^ Present address: Cavendish Laboratory Department of Physics University of Cambridge J.J. Thomson Avenue Cambridge CB3 0HE UK

**Keywords:** amplified spontaneous emission, electroluminescence, laser, light‐emitting diodes, perovskites, stimulated emission

## Abstract

The success of metal halide perovskites in photovoltaic and light‐emitting diodes (LEDs) motivates their application as a solid‐state thin‐film laser. Various perovskites have shown optically pumped stimulated emission of lasing and amplified spontaneous emission (ASE), yet the ultimate goal of electrically pumped stimulated emission has not been achieved. As an essential step toward this goal, here, a perovskite diode structure that simultaneously exhibits stable operation at high current density (≈1 kA cm^−2^) and optically excited ASE (with a threshold of 180 µJ cm^−2^) is reported. This diode structure achieves an electroluminescence quantum efficiency of 0.8% at 850 A cm^−2^, which is estimated to be ≈3% of the charge carrier population required to reach ASE in the same device. It is shown that the formation of a large angle waveguide mode and the reduction of parasitic absorption losses are two major design principles for diodes to obtain a positive gain for stimulated emission. In addition to its prospect as a perovskite laser, a new application of electrically pumped ASE is proposed as an ideal perovskite LED architecture allowing 100% external radiation efficiency.

## Graphical abstract

Optically excited amplified spontaneous emission is shown in a full stack of perovskite light‐emitting diode, designed to have an efficient waveguide mode with suppressed parasitic absorption loss. The diode also shows a stable operation at high current density of ≈1 kA cm^−2^, reaching ≈3% of the charge carrier population required for stimulated emission.

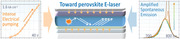

## 1 Introduction

In the last decade, metal halide perovskites have been one of the most spotlighted semiconductors for optoelectronics owing to their outstanding semiconductor properties. Stimulated by the success in highly efficient solar cells^[^ ^1^ ^]^ and light‐emitting diodes (LEDs),^[^ ^2^ ^]^ perovskite lasers are emerging as a next application of interest, with many advantages such as low‐cost fabrication and wavelength tunability,^[^ ^3^ ^]^ compared to existing solid‐state lasers. Since the first demonstration of lasing at room temperature (RT) in 2014,^[^ ^4^ ^]^ various types of perovskites have exhibited stimulated emission of lasing and amplified spontaneous emission (ASE) under optical excitation^[^ ^5^ ^]^ and recently reached a milestone of continuous‐wave (CW) lasing at low temperature or RT.^[^ ^6^ ^]^

The obvious next goal for perovskite lasers is the demonstration of electrically pumped operation of stimulated emission.^[^ ^7^ ^]^ Unlike classical bulk solid‐state lasers, the limited mobility of charge carriers in polycrystalline perovskites restricts the diode architecture to thin‐film stacks sandwiched between two fully covering electrodes. The nature of thin‐film stacks causes difficulty in electrical pumping for many complicated lasing designs with amplification along vertical or random direction, which requires significant modification in the diode structure. On the other hand, schemes such as distributed feedback (DFB) and ASE, obtaining optical gain along the lateral direction, are thought to be more compatible with thin‐film diodes. While most of the studies reporting lasing and ASE of optically excited perovskites have been performed for thin films, only a few works have demonstrated these properties on a full device stack that contains electrodes.^[^ ^5a,d,g^ ^]^ Such structures typically exhibited several shortcomings such as an order of magnitude increase in ASE threshold as compared to a thin film or significant current leakage through the DFB‐patterned interface. In addition, another challenge for laser diode operation arises from the necessity of high current densities (*J*) at high voltage. Recent studies have shown significantly reduced radiation efficiencies at such high *J*, attributed to various reasons such as Joule heating, Auger recombination, electric‐field‐induced charge separation, and imbalance.^[^ ^7^, ^8^ ^]^

Here, we demonstrate a perovskite diode that simultaneously operates at high *J* and shows ASE under optical excitation. This is an essential milestone toward an electrically pumped stimulated emission in perovskites. We use this structure to explore the prospects and application of stimulated emission in perovskite diodes and derive key design principles for future perovskite lasers.

## 2 Electroluminescence at DC and AC Current Injection

**Scheme 1:**
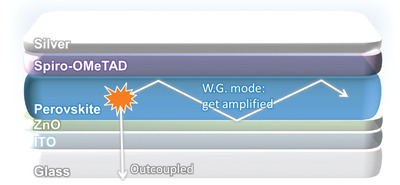
Schematic illustration of our device. Amplified spontaneous emission can occur in the waveguide mode, competing with optical losses from scattering and parasitic absorption.

**Figure 1:**
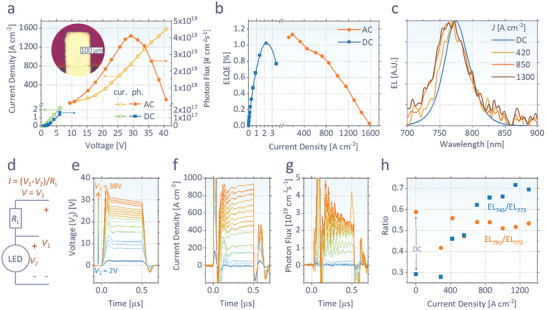
a) Current density (*J*) and outcoupled photon flux density of our perovskite diode as a function of voltage (*V*) in DC or AC. (inset: the image of the pixel used for the measurement) b) Measured ELQE and c) EL spectrum at each *J*. d) Electrical circuit used for pulsed measurement, having a load resistance (*R*_L_) between a fixed input voltage (*V*_1_) and measured device voltage (*V*_2_). e) Time‐resolved voltage, f) current density, and g) outcoupled photon flux of the device in response to various voltage inputs of 500 ns pulse. h) EL spectral ratios of 745 nm over 775 nm (EL_745_/EL_775_) and 795 nm over 775 nm (EL_795_/EL_775_) at various *J*.

Our device structure is based on glass/indium tin oxide (ITO)/polyethylenimine ethoxylated (PEIE)‐modified ZnO/Cs_0.05_FA_0.95_Pb(Br_0.1_I_0.9_)_3_ perovskite/2,2′,7,7′‐tetrakis[*N*,*N*‐di(4‐methoxyphenyl)amino]‐9,9′‐spirobifluorene (spiro‐OMeTAD) doped with Li‐ and Co‐salt/Ag, where FA indicates formamidinium, as shown in **Scheme** **1**. A near‐infrared emitting perovskite is chosen due to its low bandgap and corresponding easiness of high current injection, while Cs and Br are added to stabilize the phase.^[^ ^9^ ^]^ While our perovskite film shows high photoluminescence (PL) quantum efficiency (PLQE) of 24% (at 532 nm excitation of 160 mW cm^−2^), it is reduced to 6% upon forming a quenching interface with the spiro‐OMeTAD layer, as shown in Figure S1c in the Supporting Information. The electroluminescence (EL) quantum efficiency (ELQE) is further reduced due to the limited light extraction efficiency (LEE) and parasitic absorption loss (*A*_para_), resulting in the maximum DC ELQE of 1.0% at *J* of ≈2 A cm^−2^ (**Figure** **1**a,b). At higher current densities, above 2 A cm^−2^, the device degrades and ELQE drops rapidly. The EL spectrum peaks at 775 nm (Figure 1c) as is expected from a Cs_0.05_FA_0.95_Pb(Br_0.1_I_0.9_)_3_ perovskite.

To reach higher current densities, we reduced the area of the pixel to a dimension of 120 µm × 160 µm (precisely measured area: 1.89 × 10^−4^ cm^2^, shown in the inset of Figure 1a and Figure S3 in the Supporting Information), according to previous investigation of the ASE gain length.^[^ ^5g^ ^]^ Short voltage pulses (500 ns) are applied at a low frequency (2 Hz) to the device with a load resistance (*R*_L_ = 47 Ω) (Figure 1d). When high current is injected, thermal stability plays as a limiting factor of device performances. As shown in Figure 1e–g, at 200 ns of each pulse, the *J* starts to increase and radiation decreases at high voltage over 20 V, mainly as a result of Joule heating, which increases charge mobility, but reduces ELQE.^[^ ^8^ ^]^ The decrease of voltage after 200 ns originates from the increased voltage drop at *R*_L_ with increased current. On the other hand, for the first ≈100 ns, the voltage overshoots and the current oscillates, followed by a stabilization at 150 ns. Consequently, we average over the range 160 to 200 ns as the representative values of our AC measurements.

With a benefit of short duty cycle (10^−4^%) and small pixel area, the device can operate at high *J* injection of <1.6 kA cm^−2^ (at <40 V), without irreversible thermal damage. ELQE of 1.1% is obtained at *J* of 110 A cm^−2^, the smallest *J* with a resolvable signal in the given setup. At higher *J*, the ELQE slightly decreases and the highest photon flux of 4.1 × 10^19^ cm^−2^ s^−1^ appears at *J* = 840 A cm^−2^, where the ELQE is 0.8%. After 840 A cm^−2^, ELQE and photon flux drop more rapidly, and the pixel is destroyed at >1600 A cm^−2^.

**Figure 2:**
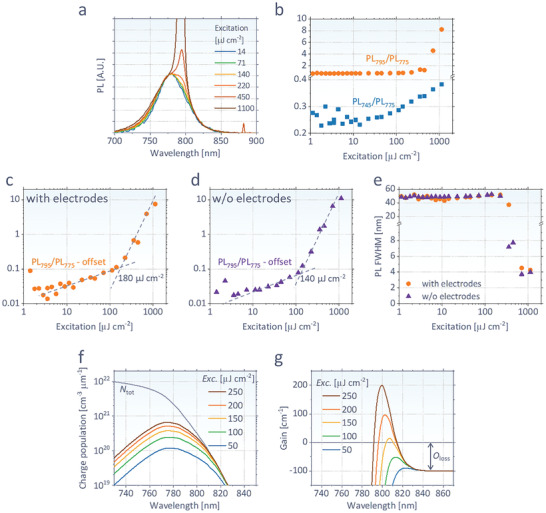
a) Normalized PL spectra of our perovskite at various excitations of a pulsed laser (1.3 ns duration, 355 nm wavelength, repetition of 10 kHz, and beam size of 5 × 10^−4^ cm^2^), fixing the intensities at 775 nm. b) PL spectral ratios of 745 nm over 775 nm (PL_745_/PL_775_) and 795 nm over 775 nm (PL_795_/PL_775_) at various excitations. c,d) PL_795_/PL_775_ subtracted by the offset value at low intensity (1 µJ cm^−2^), showing ASE threshold of 180 µJ cm^−2^ for (c) (with electrodes) and 140 µJ cm^−2^ for (d) (without electrodes). Panels (a)–(c) are measured for the full device stack, while panel (d) is for a stack without electrodes (ITO and silver). e) FWHM of PL at each excitation. f) Calculated excited charge population density (*N*_exc_) and g) gain of perovskite with a fitted joint density of states constant (*J*_0_) of 5.33 × 10^17^ cm^−2^ µm^−1^ and measured *O*_loss_ of 100 cm^−1^ from the spatially resolved PL (Figure S6, Supporting Information).

As shown in Figure 1c,h, EL spectra for higher current densities tend to be blue‐shifted, such that the EL at 745 nm rises more rapidly than the peak wavelength of 775 nm. This observation can be mainly attributed to the increased temperature of the device (Figure S4, Supporting Information). On the other hand, we could not find an evidence of ASE at the given range of electrical pumping. At 795 nm, where ASE appears following the optical excitation (to be shown in **Figure** **2**), there is no additional peak appearing at high excitation (Figure 1c,h).

It is notable that our device deviates from the typical design rules for conventional LEDs. State‐of‐the‐art perovskite LEDs reported to date mostly have i) low trap recombination, ii) suppressed interface quenching, iii) thin emitter (<100 nm) with a microcavity optimized for forward emission and suppressed waveguide mode, and iv) (optionally) rough interfaces to enhance the outcoupling.^[^ ^2^, ^10^ ^]^ However, those rules are largely not applicable to lasing diodes with a lateral gain direction. In 3D perovskites like the one used in our devices, the lifetime of bimolecular radiative recombination rapidly decreases at high charge carrier concentrations. Then, the contribution from monomolecular trap recombination or charge diffusion to the quenching site becomes relatively small. Hence, the ELQE at high *J* becomes almost independent of (i) and (ii), and rather limited by other losses such as nonradiative bimolecular recombination and Auger recombination.^[^ ^11^ ^]^ For example, our AC ELQE of 0.8% at 840 A cm^−2^ is very similar to the previously reported values of ≈1% at 200–1000 A cm^−2^ in earlier works, which used efficient LEDs with an order of magnitude higher ELQE (>10%) than ours at low DC current injection. Moreover, instead of (iii) and (iv) for forward emission, our device is designed to have an efficient waveguide mode suppressing optical loss, as will be discussed in the latter section for optical modeling.

## 3 Optically Pumped Amplified Spontaneous Emission

Figure 2 and Figure S5 in the Supporting Information show the results of optically excited ASE in the devices described in the previous section. At each excitation, the PL is normalized to the peak intensity at 775 nm (PL_775_). As shown in Figure 2a,b, an additional peak near 795 nm (PL_795_) begins to arise under strong pulsed laser excitation, as an evidence of ASE. In Figure 2c, we subtract a constant offset (= a value of PL_775_/PL_795_ at 1 µJ cm^−2^) from PL_775_/PL_795_ to better distinguish the peak in a logarithmic scale. It is notable that PL_795_/PL_775_ shows an increasing trend even at weak excitations below 100 µJ cm^−2^. That can be attributed to the contribution from photons in the waveguide mode, which are red‐shifted from the initial PL due to larger perovskite reabsorption at short wavelengths.^[^ ^5g^ ^]^ Outcoupling of those photons via scattering contributes to the long wavelength shoulder of total PL spectrum. At higher (but below the ASE threshold) excitation, the net absorption cross‐section of perovskite at long wavelengths decreases due to the reduced charge concentration at ground state. Consequently, more photons in the waveguide mode are collected with smaller reabsorption loss, resulting in the rise of relative PL_795_. However, such increasing trend of PL_795_ at low excitation is clearly distinguishable from that at ASE regime with a large slope of PL_795_/PL_775_ at high excitation. The ASE threshold, at which point the slope is changed and photon amplification starts, is determined to be 180 µJ cm^−2^, as is shown by the fitting lines in Figure 2c.

The increase of the PL at the short wavelengths (e.g., PL_745_) shown in the electrical excitation also appears upon optical excitation (Figure 2b). However, the increasing slope is much smaller for the optical excitation, despite the higher density of excited charge carriers near the ASE threshold. This indicates that the effect of thermal stress is more severe upon electrical excitation, due to its long pulse duration (500 ns) and high voltage (10–40 V) delivering more heat than the laser source used for the optical excitation, whose duration is only 1.3 ns with a wavelength of 355 nm (3.5 eV photon energy). This difference in thermal stress exemplifies why electrical pumping is more challenging than optical pumping in laser application.

The similar trend of ASE is observed for a stack without electrodes (Figure 2d), with a lower threshold of 140 µJ cm^−2^. It is notable that, unlike a previous work that reported an order of magnitude increased ASE threshold with a metal electrode,^[^ ^5g^ ^]^ here the threshold in a full device is only 30% higher than that without electrodes. This result indicates that the waveguide mode is well confined in a perovskite layer having a large refractive index (*n* ≈ 2.5), without significant optical loss from electrodes, as will be proven by optical modeling in the next section. For both samples with and without electrodes, full‐width half‐maximum (FWHM) of PL decreases from ≈50 nm (dominated by spontaneous emission) to ≈4 nm (dominated by ASE) as excitation increases.

Figure 2f,g provides optical insights for device design through the calculation of population density of excited charge carriers (*N*_exc_) at various excitation intensities, and gain of ASE at each wavelength (*λ*) based on their relation^[^ ^5g^ ^]^

(1) $$\mathrm{Gain}\left(\lambda \right)\;=\;\alpha_{\mathrm{act}}\left(\lambda \right)\times \frac{2N_{\mathrm{exc}}\left(\lambda \right)-N_{\mathrm{tot}}\left(\lambda \right)}{N_{\mathrm{tot}}\left(\lambda \right)}-\alpha_{\mathrm{para}}\left(\lambda \right)-O_{\mathrm{loss}}\left(\lambda \right)$$

where *α*_act_ and *α*_para_ are the absorption coefficients of the perovskite and parasitic layers during waveguide propagation, respectively. *O*_loss_ is an additional optical loss caused by, among other reasons, scattering and quantified to be 100 cm^−1^ in our perovskite (assumed wavelength‐independent), as shown by spatially resolved PL (Figure S6, Supporting Information). The total joint density of states (*N*_tot_) is assumed to be proportional to the absorption coefficient of the perovskite with a proportionality constant of *J*_0_.^[^ ^5g^ ^]^ In the electrode‐free stack, by assuming zero *α*_para_, *J*_0_ can be obtained by fitting the calculated threshold (i.e., excitation for gain = 0) to the measured value of 140 µJ cm^−2^, resulting in *J*_0_ = 5.33 × 10^17^ cm^−2^ µm^−1^. *N*_exc_ is obtained by recursively filling the vacant states according to the Boltzmann distribution, while potential charge recombination loss during excitation is not taken into account.^[^ ^5g^ ^]^ At short wavelengths, the upper bound gain is larger due to large *α*_act_, however, a stronger excitation is required to fill the states and reach a population inversion. On the other hand, at long wavelengths, although the states can be easily filled at weak excitation, *α*_act_ is too small to exceed *α*_para_ + *O*_loss_ even at perfect population inversion (*N*_exc_ = *N*_tot_). Hence, *α*_para_ and *O*_loss_ are primary parameters to determine the ASE threshold (i.e., excitation to achieve a positive gain) of a given system. Such spectral trade‐off confines ASE spectrum to a specific range, e.g., 790–800 nm in our device, and causes a blue‐shift of ASE peak at higher excitations as is shown in Figure 2a,g.

## 4 Optical Analysis for Device Optimization

**Figure 3:**
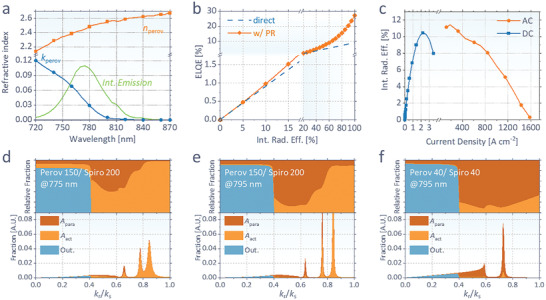
a) Refractive index (*n* + *ik*) and internal emission spectrum of our Cs_0.05_FA_0.95_Pb(Br_0.1_I_0.9_)_3_ perovskite used for the calculation. b) Calculated ELQE including and excluding photon recycling effect as a function of *η*_rad_, for our device structure of glass/ITO 150 nm/PEIE‐modified ZnO 40 nm/perovskite 150 nm/Li, Co‐doped spiro‐OMeTAD 200 nm/Ag. c) *η*_rad_ from the measured ELQE versus *J*. d–f) Fractions of optical energy (*A*_para_, *A*_act_, and outcoupled) as a function of relative radial propagation vector (*k*_r_/*k*_s_), in our device structure at a wavelength of d) 775 nm or e) 795 nm, or a structure having thinner perovskite and spiro‐OMeTAD layers (40 nm thick for both) at f) 795 nm.

The successful demonstration of ASE in our diodes can be attributed to the dedicated optical design. While classical methods of optical modeling for LEDs are based on the assumption of nonabsorbing emissive layer, they suffer from optical divergence when absorption (or inverse absorption in lasers) of the emissive layer is taken into account, thus restricting the analysis for thin‐film lasing diodes. Here, we resolve this divergence by assuming recycling of near‐field self‐coupled dipoles, in a way recently presented.^[^ ^10^, ^12^ ^]^ The inclusion of reabsorption by the perovskite layer allows to analyze not only the propagation modes formed in the device, but also the ratio of absorptions by perovskite and parasitic layers, which is critical in determining the optical loss of stimulated emission, and yet has not been investigated before. **Figure** **3**a shows the refractive index of the perovskite layer obtained from the measured transmission and reflection (Figure S7, Supporting Information). The internal emission spectrum is reversely obtained from the measured external EL spectrum and the calculated outcoupling efficiency at each wavelength.

Figure 3b shows the calculated ELQE as a function of internal radiation efficiency (*η*_rad_). Since our device is not optimized for forward emission, direct outcoupling efficiency is relatively small (9.2%). Aided by the photon recycling effects,^[^ ^10^, ^12^, ^13^ ^]^ the maximum achievable ELQE is shown to reach 26%. However, in the range of ELQE < 1.1%, which we experimentally achieved, the contribution of photon recycling is shown to be not significant. Using this ELQE–*η*_rad_ relationship, the measured ELQE in Figure 1b can be converted to *η*_rad_ at each *J*, as shown in Figure 3c. *η*_rad_ is shown to be 11% at the maximum point and drops at higher *J*, reaching 8.1% at 840 A cm^−2^, where the maximum photon flux is observed.

Figure 3d–f shows the fraction of dipole energy as a function of radial propagation vector (*k*_r_/*k*_s_ = sin *φ*, *φ*: emission angle inside the emitter) in the range of the radiative mode. Due to a thick optical spacer (spiro‐OMeTAD), the nonradiative mode (*k*_r_/*k*_s_ > 1) is calculated to be small in the given device structure. The energy at each mode can be eventually outcoupled or reabsorbed by the perovskite (*A*_act_) or parasitic layers (*A*_para_). In our device structure (Figure 3d,e), two strong peaks of waveguide mode appear at large *k*_r_/*k*_s_ of 0.76 and 0.84 for both wavelengths of 775 and 795 nm. Since the refractive index of the perovskite (*n*_perov_ ≈ 2.5) is sufficiently larger than those for nearby layers of spiro and ZnO (*n* ≈ 1.6), photons with a large emission angle (*k*_r_/*k*_s_ > 1.6/2.5 = 0.8) are confined in the perovskite layer during lateral propagation. Consequently, while the relative fraction of *A*_para_ at *k*_r_/*k*_s_ of 0.76 is still significant, it is shown to be suppressed to 3% and 11% at *k*_r_/*k*_s_ of 0.84 for 775 and 795 nm, respectively. Therefore, the mode at *k*_r_/*k*_s_ of 0.84 can be considered as a major pathway for photon amplification when ASE occurs. It is notable that *A*_para_ is still nonzero at such large *k*_r_/*k*_s_, due to the skin‐depth absorption at electrodes during waveguide propagation. Here, the absorption from Ag is mostly prevented by our 200 nm thick spiro‐OMeTAD layer, hence *A*_para_ at large *k*_r_/*k*_s_ mainly comes from the ITO absorption.

Unlike our device, many of the recent perovskite LEDs have thinner perovskite active layers and optical spacers, to maximize the forward emission. Figure 3f shows the analysis result for such a device at 795 nm, assumed to have a 40 nm thick perovskite and 40 nm thick optical spacer (spiro‐OMeTAD). Compared to Figure 3e at the same wavelength, the area of outcoupling mode (sky blue) is enlarged, having 2.4 times enhanced direct outcoupling efficiency of 22.3% (without photon recycling). However, the thinner emissive layer eliminates the mode at *k*_r_/*k*_s_ of 0.84, which is confined in the perovskite. Moreover, *A*_para_ dominates over *A*_act_ even at such large *k*_r_/*k*_s_, because of thinner optical spacer, which is not sufficient to prevent the Ag absorption, and thinner perovskite, which causes more bounces of photons at the interfaces during propagation. In such a structure, the gain in Equation (1) is always negative (*α*_act_ < *α*_para_) regardless of pumping intensity, hence stimulated emission is intrinsically not achievable. Further results of our modeling are available in Figures S8 and S9 in the Supporting Information.

## 5 Prospect of Electrically Pumped ASE and Laser in Perovskite Diodes

**Figure 4:**
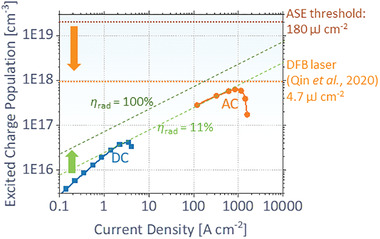
Estimated free charge population (*N*_exc_) under various DC or AC current injections. Two dotted lines indicate the thresholds of ASE achieved in our device and DFB laser reported by Qin et al.,^[^ ^6a^ ^]^ respectively. Two dashed lines indicate the expected *N*_exc_ for constant *η*_rad_ of 11% and 100%, respectively. The arrows represent the desired directions of enhanced *η*_rad_ (green) and reduced threshold (orange), to achieve the electrically pumped stimulated emission.

**Figure****4** displays the calculated free charge carrier concentration (*N*_exc_) excited by optical or electrical pumping. The ASE threshold of 180 µJ cm^−2^ corresponds to *N* = 2.0 × 10^19^ cm^−3^, by dividing it by photon energy and film thickness (*T*), and considering measured reflectance (5%) of the device at excitation wavelength. Here, we simplified calculation by neglecting the recombination loss during excitation. In case that recombination loss affects optical pumping, our calculated value of *N* can be understood as an upperbound of the threshold charge population.

*N*_exc_ under electrical pumping is calculated by assuming the steady‐state and bimolecular radiative recombination

(2) $$\frac{dN_{\mathrm{exc}}}{dt}=\;G-R\;=\frac{J}{qT}\;-\;\frac{B_{\mathrm{rad}}N_{\mathrm{exc}}^{2}}{\eta_{\mathrm{rad}}}=\;0$$

where *q* = 1.6 × 10^−19^ C, the bimolecular radiative recombination constant (*B*_rad_) is assumed to be 7 × 10^−11^ cm^3^ s^−1^,^[^ ^11^ ^]^ and *η*_rad_ is taken from Figure 3c. The highest *N*_exc_ value of 6.4 × 10^17^ cm^−3^ is obtained at *J* = 840 A cm^−2^ where the maximum photon flux appears in Figure 1a. Hence, electrical pumping for our device reaches 3% of charge population required for stimulated emission in the same device.

Our results illustrate the bright prospect of electrically pumped stimulated emission in perovskites to be reached in the near future. While our device consists of a Cs_0.05_FA_0.95_Pb(Br_0.1_I_0.9_)_3_ perovskite with an ASE threshold of 140 µJ cm^−2^ (without electrodes), there have been several recent reports for lasing in perovskites, having lower threshold.^[^ ^5^, ^6^ ^]^ For example, a DFB laser recently succeeding in CW operation showed a pulsed lasing threshold of 4.7 µJ cm^−2^,^[^ ^6a^ ^]^ corresponding to *N*_exc_ = 9.6 × 10^17^ cm^−3^ close to what our electrical pumping reaches. DFB lasing can be implemented by using a patterned substrate or nanoimprinting the emissive layer^[^ ^5a,d^ ^]^ when a device has a stable high current operation.

The efforts to enhance *η*_rad_ are also necessary to suppress the loss of excited charges upon electrical pumping. While the population‐independent loss such as trap recombination can be easily dominated over by fast bimolecular recombination at high population, it is necessary to further investigate the loss pathways responsible for efficiency drop at high *J*, such as Joule heating and Auger recombination, which have been less spotlighted in the field of LEDs. Especially, proper thermal management, such as the use of heat spreaders,^[^ ^5^, ^8^ ^]^ is required not only for enhancing *η*_rad_, but also for preventing the increase of the ASE or lasing threshold, which is known to be sensitive to changes in temperature.^[^ ^5^, ^6^ ^]^

## 6 Application for LEDs with Unity Outcoupling Efficiency

**Figure 5:**
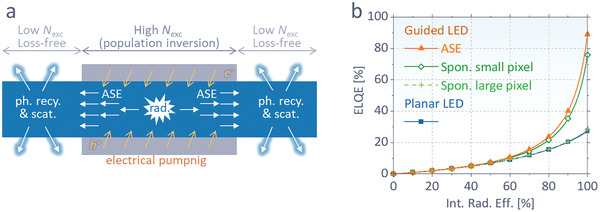
a) Schematic of the proposed architecture utilizing ASE for 100% outcoupling efficiency. The device achieves population inversion through electrical pumping and delivers photons to a region out of the pixel through ASE with little *A*_para_. The photons can be outcoupled through recursive events of photon recycling or scattering in the region out of the pixel. b) Expected ELQEs for the proposed structures with a waveguided photon delivery through ASE or spontaneous emission, compared to a conventional planar LED. The guided structure without ASE is effective only for unrealistically small (submicrometer scale) pixel sizes.

In addition to a laser diode, we here propose another future application of electrically pumped ASE, that is, a perovskite LED with a unity outcoupling efficiency. As is shown in Figure 3b and previous literature,^[^ ^10^, ^12^ ^]^ although photon recycling can break the trapped modes and help light extraction, the maximum outcoupling efficiency of a perovskite LED is still limited to ≈30% due to *A*_para_ competing with outcoupling during the recursive events of photon recycling. As a scheme to avoid such loss from *A*_para_, previous research proposed an architecture having reduced electrode area, where photons in a waveguide mode are recycled into regions out of the pixel area, not covered by electrodes.^[^ ^12^ ^]^ However, according to the reported calculation, which will also be shown in **Figure** **5**b, the scheme based on spontaneous emission is effective only when the electrode dimensions are in a submicrometer scale.^[^ ^12^ ^]^ For larger pixel widths (10^−2^–10^0^ mm), even photons in waveguide mode still suffer from *A*_para_ loss before escaping the pixel through 1) the propagation modes with insufficiently large *k*_r_/*k*_s_, e.g., those smaller than 0.8 in Figure 3d–f, and 2) recursive photon recycling events during propagation, which causes recursive loss from *A*_para_. Such aspects have made this scheme unrealistic.

In this regard, electrically pumped ASE, if achieved in the near future, can provide a pathway for efficient photon emission beyond the aforementioned limitations, as depicted in Figure 5a. When a population inversion is made by strong electrical pumping, excited electrons coherently decay through ASE. Although ASE brings a fast radiative decay (≈10^−11^ s),^[^ ^5f^ ^]^ which dominates over other loss pathways, it does not directly assist external radiation as all radiation is trapped in the perovskite. Instead, unlike spontaneous emission, ASE confines all photons into a specific mode (e.g., *k*_r_/*k*_s_ of 0.84 at wavelength of 795 nm in our device) with small (ideally zero) *A*_para_, hence efficiently delivers them to the outside regardless of pixel size. At the outside region where population inversion ends, the photons can be outcoupled through reabsorption and reemission (i.e., photon recycling) or light scattering. Since the region is free from electrode absorption, photons can be recursively recycled or scattered without *A*_para_ until outcoupled. In this way, the upper bound of classical LED outcoupling efficiency can be broken. It is notable that while ASE can easily dominate over trap losses inside the pixel with a high excitation, we still need a low‐trap (ideally trap‐free) perovskite to reach such high efficiency, as photons are recycled at a region with low excitation, as shown in Figure 5b. The calculated ELQE of the proposed scheme reaches a maximum ELQE of 89%, much above those for spontaneous emission‐based devices (refer to the Experimental Section for details of calculation), not to mention the planar device. The loss of 11% comes from *A*_para_ in the guided mode in Figure 3e, which can be further suppressed by further separating the ITO and the perovskite (i.e., a thicker electron injecting layer, ZnO) to reach unity ELQE in the future.

## 7 Conclusion

We achieve a strong electrical pumping (ELQE 0.8% at 840 A cm^−2^) and optically excited ASE (with a threshold of 180 µJ cm^−2^) simultaneously in a single perovskite diode, as an essential milestone toward electrically pumped perovskite lasers. Formation of waveguide mode at a large propagation angle and the suppression of parasitic absorption losses are shown to be the two major design rules to achieve a positive optical gain in the lateral direction. Our device is estimated to reach 3% of the free charge population density required for ASE, showing that electrically pumped perovskite laser is already within the achievable range. We also present a future application of electrically pumped ASE as an ideal perovskite LED configuration capable to reach 100% ELQE.

## 8 Experimental Section

### Preparation of Perovskite Solution

First, 73.8 mg PbBr_2_ (Sigma‐Aldrich) and 382.5 mg PbI_2_ (TCI) were dissolved in 906 µL solvent of dimethylformamide:dimethyl sulfoxide (DMF:DMSO) mixed in 4:1 v/v ratio, on a hot plate of 110 ℃. After cooling it down to room temperature, 872 µL of this solution was added to 150 mg FAI (Lumtec), together with 43.6 µL of CsI 1 m solution (Sigma‐Aldrich, 256 mg mL^−1^ in DMSO), targeting 1 m Cs_0.05_FA_0.95_Pb(Br_0.1_I_0.9_)_3_ solution. The solution was diluted to 0.38 m by adding more 4:1 DMF:DMSO solvent.

### Device Fabrication

Glass/ITO substrates (1.2 cm × 1.2 cm) were sequentially cleaned by detergent, deionized water, acetone, and isopropyl alcohol with sonication (10 min each). The substrates were dried by N_2_ blowing and cleaned with an oxygen plasma at 100 mW for 10 min. ZnO solution was prepared by dissolving 0.46 m zinc acetate dehydrate, 0.46 m ethanolamine in 2‐methoxyethanol and it was stringed at 70 ℃ for 24 h, and spin‐coated on the ITO substrates at 2000 rpm for 60 s.^[^ ^14^ ^]^ ZnO layer was partially removed by mechanical scratch to form an ITO contact area, and then annealed at 300 ℃ for 30 min. ZnO surface was modified by spin‐coating PEIE solution (0.05 wt% in isopropyl alcohol) at 5000 rpm for 30 s and annealing at 100 ℃ for 10 min.^[^ ^2^, ^15^ ^]^ Then, the substrates were moved to a N_2_‐filled box. 0.38 m Cs_0.05_FA_0.95_Pb(Br_0.1_I_0.9_)_3_ perovskite solution was spin‐coated at 1000 rpm for 10 s followed by 4000 rpm for 25 s. 5 s before the end of the whole spin‐coating process, 200 µL toluene was carefully (i.e., not too fast) dripped onto the substrate as an antisolvent treatment.^[^ ^9a^ ^]^ Refer to Figure S1a,b in the Supporting Information for comparing PLQEs of the films in various stoichiometry and antisolvents. The samples were annealed at 100 ℃ for 30 min, resulting in a 150 nm thick perovskite film (measured using a Dektak 150 surface profiler). As a hole transporting layer, Li‐ and Co‐doped spiro‐OMeTAD (spiro‐OMeTAD 72.3 mg, TBP 28.8 µL, bis(trifluoromethane)sulfonimide lithium salt solution (Li‐TFSI, 500 mg mL^−1^ in acetonitrile) 16.8 µL, and FK209 Co(III)‐TFSI solution (500 mg mL^−1^ in acetonitrile) 30.2 µL in chlorobenzene 1 mL) was spin‐coated at 4000 rpm for 30 s without further annealing process, resulting in a 200 nm thick film (measured using a Dektak 150 surface profiler). The region for ITO contact was removed by scratch. The device was finalized by thermally evaporating 80 nm thick Ag electrode with a patterned mask for small pixel area. Pixel area (1.89 × 10^−4^ cm^2^) was measured using an optical microscope. All characterization was performed in the air without encapsulation.

### Electroluminescence Characterization

DC ELQE was measured by a calibrated silicon photodiode (Thorlabs, FDS10X10), which was directly attached to the device. Two separate source measure units (SMUs) were used for applying voltage to the device and reading photocurrent from the photodiode, respectively. For AC measurement, a pulse generator (HP 8114A) applied voltage pulses (2 Hz, 500 ns duration) with a load resistance (*R*_L_) of 47 Ω. EL was measured using a biased Si photodiode (Thorlabs, DET100A2, rise time of 35 ns), connected to a current amplifier (FEMTO, DHPCA‐100) with a set gain of 10^3^ V A^−1^ and bandwidth of 200 MHz. Considering a limited diameter of photodiode (9.8 mm) and its distance to the emissive device (4.4 mm), a correction factor was applied to the measured EL flux (assuming Lambertian emission). Voltages applied to before and after *R*_L_ (*V*_1_ and *V*_2_, respectively) and output of the current amplifier were read by an oscilloscope (Rohde&Schwarz HMO3004). To avoid the permanent damage to the device, the measurement with a photodiode was performed over the limited *V*_2_ range of 0–30 V. Then, the device was moved to the spectrometer setup and *V*_2_ was swept over 0–40 V (*V*_1_ of 0–70 V). EL spectrum was measured at each voltage using a spectrometer equipped with an array of cooled charge‐coupled devices (CCDs), with a long integration time of 20 s to compensate the low duty cycle of the pulse. The photon flux at *V*_2_ > 30 V was plotted using the area of EL spectrum, which was calibrated with the data using a photodiode at the same condition. The SMUs and oscilloscope were controlled with the software SweepMe! (sweep‐me.net).

### ASE Characterization

ASE property of the device was characterized with a focused pulse laser excitation (wavelength: 355 nm; duration: 1.3 ns; repetition rate: 10 kHz).^[^ ^5g^ ^]^ The beam size was set to 5 × 10^−4^ cm^2^ by slightly defocusing the convex lens. The excitation intensity was swept from low to high by inserting proper optical filters. Photons were collected at the opposite side from the excitation, using a high NA objective lens and spectrometer with an array of cooled CCDs.

### Optical Analysis

The modeling was performed with a recently proposed method.^[^ ^10^, ^12^ ^]^ Poynting vectors at each interface were calculated using transfer‐matrix formalism (TMF). *A*_act_ and *A*_para_ were obtained from the difference in Poynting vectors of front and back interfaces of each layer. Near‐field self‐coupling in perovskite was assumed to be fully recycled. Diode structure was set to be glass/ITO 150 nm/PEIE‐modified ZnO 40 nm/perovskite 150 nm/Li, Co‐doped spiro‐OMeTAD 200 nm/Ag. Refractive index for perovskite was obtained by fitting the measured transmission and reflection (Figure S7, Supporting Information), and that for other layers were from literature.^[^ ^16^ ^]^ The results were integrated by sweeping the conditions of wavelength, *k*_r_/*k*_s_, orientation (*x*, *y*, and *z*‐oriented dipoles), polarization (transverse electric (TE) and transverse magnetic (TM) modes), and dipole position (20 positions uniformly distributed over a perovskite layer). For ELQE calculation, charge balance efficiency^[^ ^12^ ^]^ was assumed to be 100% as ZnO (valence band at ≈7.7 eV) and spiro‐OMeTAD (conduction band at ≈2.2 eV) had sufficient energy barrier to holes and electrons in the perovskites, respectively.^[^ ^17^ ^]^

### ELQE Estimation for the Novel LED Structure

In the given LED structure of glass/ITO 150 nm/PEIE‐modified ZnO 40 nm/perovskite 150 nm/Li, Co‐doped spiro‐OMeTAD 200 nm/Ag, when spontaneous emission occurred, 9.1% was outcoupled, 66.8% was absorbed by perovskite (*A*_act_), and 24.1% was absorbed by parasitic layer (*A*_para_) according to the above‐mentioned TMF calculation. In the proposed novel LED structure with a small pixel area, it was assumed that pixel width was comparable to perovskite reabsorption length, hence all the photons of *A*_act_ were recycled in the region out of the pixel with an efficiency of *η*_rad_. On the other hand, for the large pixel size (an order longer than the reabsorption length), photons were assumed to undergo ten times of recursive photon recycling inside the pixel, where *A*_para_ loss occurred per recycling event, before escaping the pixel. For the structure with ASE, all photons were assumed to be confined in the mode of *k*_r_/*k*_s_ of 0.84 and wavelength of 795 nm, where *A*_para_ was 11%. With a perfect population inversion, the fraction of *A*_act_ (89%) corresponded to the inverse absorption, and it was delivered to outside without undergoing further photon recycling or optical loss in the pixel, achieving maximum ELQE of 89% in the radiative limit.

### Statistical Analysis

In Figure 1a,b, *V*, *J*, and ELQE at each AC condition were determined by averaging pulse responses between 160 and 200 ns shown in Figure 1e–g, as described in the main text. To collect EL and PL at various wavelengths in Figures 1h and 2b–d, the spectra within ±5 nm were averaged per wavelength. Each PL in Figure 2a was normalized by a value at 775 nm. ASE thresholds in Figure 2c,d were fitted from the cross of two asymptotic lines (in log–log scale) at regions of low and high excitations, containing 5 points per decade of excitation.

## Conflict of Interest

Dr. Axel Fischer is cofounder of “Axel Fischer und Felix Kaschura GbR,” which provided the measurement software “SweepMe!” (sweep‐me.net).

## Supporting information

Supporting Information

## Data Availability

The data that support the findings of this study are available from the corresponding author upon reasonable request.

## References

[1] a) A. Kojima, K. Teshima, Y. Shirai, T. Miyasaka, J. Am. Chem. Soc. 2009, 131, 6050; 19366264 10.1021/ja809598r

[2] a) Z. K. Tan, R. S. Moghaddam, M. L. Lai, P. Docampo, R. Higler, F. Deschler, M. Price, A. Sadhanala, L. M. Pazos, D. Credgington, F. Hanusch, T. Bein, H. J. Snaith, R. H. Friend, Nat. Nanotechnol. 2014, 9, 687; 25086602 10.1038/nnano.2014.149

[3] K. P. Goetz, A. D. Taylor, F. Paulus, Y. Vaynzof, Adv. Funct. Mater. 2020, 30, 1910004.

[4] a) F. Deschler, M. Price, S. Pathak, L. E. Klintberg, D.‐D. Jarausch, R. Higler, S. Hüttner, T. Leijtens, S. D. Stranks, H. J. Snaith, M. Atatüre, R. T. Phillips, R. H. Friend, J. Phys. Chem. Lett. 2014, 5, 1421; 26269988 10.1021/jz5005285

[5] a) Y. Jia, R. A. Kerner, A. J. Grede, A. N. Brigeman, B. P. Rand, N. C. Giebink, Nano Lett. 2016, 16, 4624; 27331618 10.1021/acs.nanolett.6b01946

[6] a) C. Qin, A. S. D. Sandanayaka, C. Zhao, T. Matsushima, D. Zhang, T. Fujihara, C. Adachi, Nature 2020, 585, 53; 32879501 10.1038/s41586-020-2621-1

[7] a) A. S. D. Sandanayaka, T. Matsushima, F. Bencheikh, S. Terakawa, W. J. Potscavage, C. Qin, T. Fujihara, K. Goushi, J.‐C. Ribierre, C. Adachi, Appl. Phys. Express 2019, 12, 061010;

[8] a) H. Kim, L. Zhao, J. S. Price, A. J. Grede, K. Roh, A. N. Brigeman, M. Lopez, B. P. Rand, N. C. Giebink, Nat. Commun. 2018, 9, 4893; 30459326 10.1038/s41467-018-07383-8 PMC6244086

[9] a) A. D. Taylor, Q. Sun, K. P. Goetz, Q. An, T. Schramm, Y. Hofstetter, M. Litterst, F. Paulus, Y. Vaynzof, Nat. Commun. 2021, 12, 1878; 33767163 10.1038/s41467-021-22049-8 PMC7994557

[10] a) L. Zhao, K. M. Lee, K. Roh, S. U. Z. Khan, B. P. Rand, Adv. Mater. 2019, 31, 1805836; 10.1002/adma.201805836 30412319

[11] J. M. Richter, M. Abdi‐Jalebi, A. Sadhanala, M. Tabachnyk, J. P. H. Rivett, L. M. Pazos‐Outon, K. C. Godel, M. Price, F. Deschler, R. H. Friend, Nat. Commun. 2016, 7, 13941. 28008917 10.1038/ncomms13941 PMC5196482

[12] C. Cho, B. Zhao, G. D. Tainter, J.‐Y. Lee, R. H. Friend, D. Di, F. Deschler, N. C. Greenham, Nat. Commun. 2020, 11, 611. 32001711 10.1038/s41467-020-14401-1 PMC6992794

[13] L. M. Pazos‐Outon, M. Szumilo, R. Lamboll, J. M. Richter, M. Crespo‐Quesada, M. Abdi‐Jalebi, H. J. Beeson, M. Vrucinic, M. Alsari, H. J. Snaith, B. Ehrler, R. H. Friend, F. Deschler, Science 2016, 351, 1430. 27013728 10.1126/science.aaf1168

[14] S. Kwon, K.‐G. Lim, M. Shim, H. C. Moon, J. Park, G. Jeon, J. Shin, K. Cho, T.‐W. Lee, J. K. Kim, J. Mater. Chem. A 2013, 1, 11802.

[15] C. Bao, W. Xu, J. Yang, S. Bai, P. Teng, Y. Yang, J. Wang, N. Zhao, W. Zhang, W. Huang, F. Gao, Nat. Electron. 2020, 3, 156. 32226921 10.1038/s41928-020-0382-3 PMC7100905

[16] a) C. Cho, J. Y. Lee, Opt. Express 2013, 21, A276; 23482290 10.1364/OE.21.00A276

[17] B.‐T. Liu, B.‐W. Guo, R. Balamurugan, Nanomaterials 2020, 10, 1753. 10.3390/nano10091753 PMC7559937 32899846

