# Supplementary material for: Electrical Pumping of Perovskite Diodes: Toward Stimulated Emission
Source: Adv Sci (Weinh). 2021 Jul 8;8(17):2101663. doi: 10.1002/advs.202101663 (PMC8425921; doi:10.1002/advs.202101663)
Supplement: Supplementary file 1 — Supporting Information [file ADVS-8-2101663-s001.pdf]

WILEY-VCH

© 2021 Wiley-VCH GmbH

ADVANCED SCIENCE
Open Access

Supporting Information

for *Adv. Sci.*, DOI: 10.1002/advs.202101663

Electrical Pumping of Perovskite Diodes: Towards Stimulated Emission

Changsoon Cho, Tobias Antrack, Martin Kroll, Qingzhi An, Toni R. Bärschneider, Axel Fischer, Stefan Meister, Yana Vaynzof, Karl Leo**

Supporting Information

Electrical Pumping of Perovskite Diodes: Towards Stimulated Emission

Changsoon Cho, Tobias Antrack, Martin Kroll, Qingzhi An, Toni R. Bärschneider, Axel Fischer, Stefan Meister, Yana Vaynzof, Karl Leo**

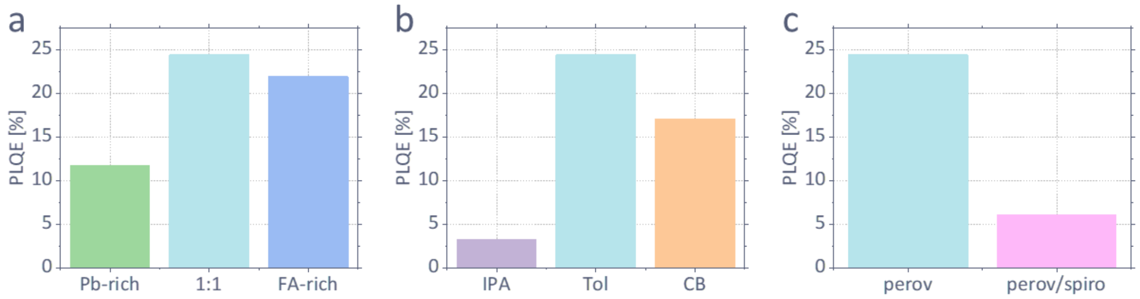

Figure S1. PLQE of $Cs_{0.05}FA_{0.95}Pb(Br_{0.1}I_{0.9})_3$ perovskite in various conditions. a) Varying ratio between $Cs_{0.05}FA_{0.95}$ and $Pb(Br_{0.1}I_{0.9})_3$ in precursor solution (1:1.05 for Pb-rich, 1.05:1 for FA-rich). b) Varying solvents for antisolvent process. c) Perovskites with and without spiro-OMeTAD coated, as a hole quenching layer. All perovskite films are coated on glass/ ITO/ PEIE-modified ZnO. Precursor ratio of 1:1 and antisolvent using Toluene are default condition. PLQE is characterized in the intergrating sphere, at the 532 nm excitation of 160 mW cm^{-2}.

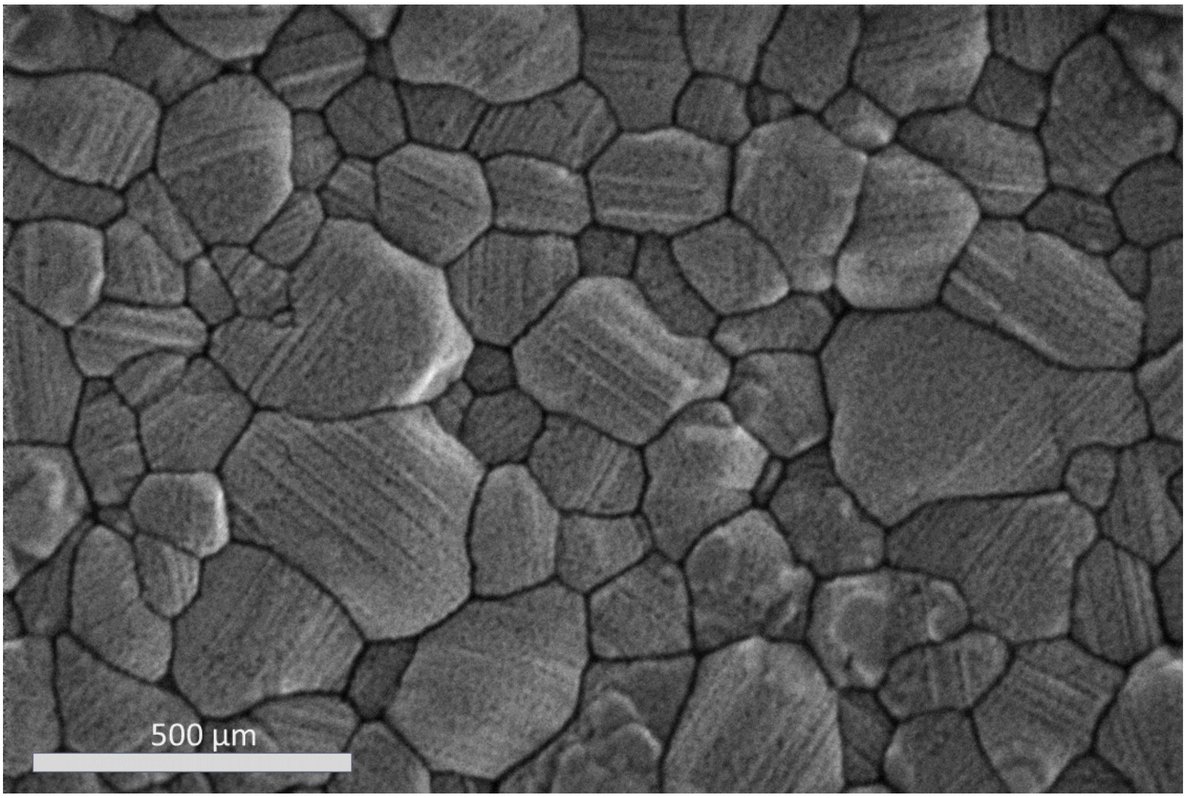

Figure S2. Scanning electron microscope (SEM) image of $Cs_{0.05}FA_{0.95}Pb(Br_{0.1}I_{0.9})_3$ perovskite coated on glass/ ITO/ PEIE-modified ZnO.

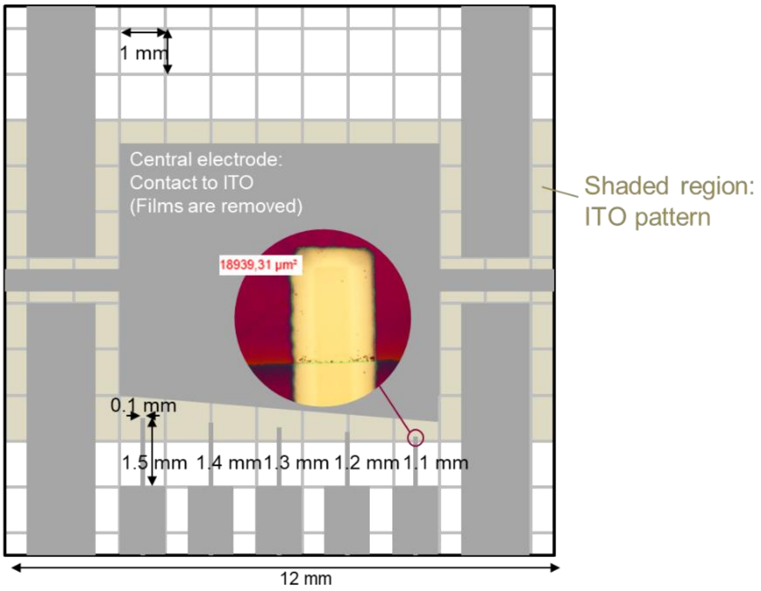

Figure S3. The electrode configuration used to make small and large pixel areas on the same substrate. Each grid represents 1 mm. Active pixel is formed at the intersection of ITO (shaded stripe at the center) and Ag (grey region) electrodes. 4 pixels with an area of 0.045 cm^2 are used for DC measurement and ASE characterization. Ag stripes with a designed width of 0.1 mm and various lengths are implemented to make a small pixel area. ITO contact is made with the central electrode by removing the films in that region. Measured area of the smallest pixel is 0.000189 cm^2 as shown in the inset.

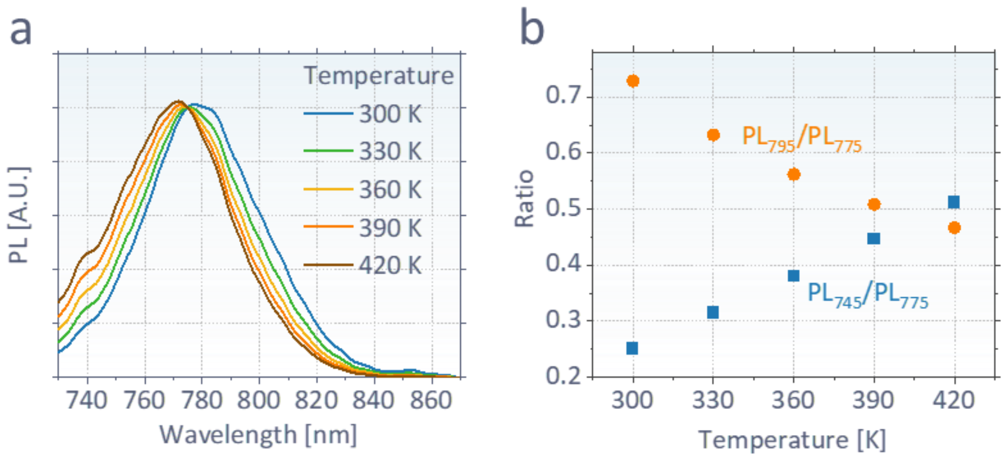

Figure S4. a) Calculated PL spectra for various temperatures. b) PL spectral ratios of 745 nm over 775 nm (PL_{745}/PL_{775}) and 795 nm over 775 nm (PL_{795}/PL_{775}) at various temperatures. PL spectrum at each temperature is obtained by assuming Boltzmann distribution of excited charge carriers, in the same way as Figure 2f. Changes in bandgap and DOS spectrum for changed temperature are not taken into account.

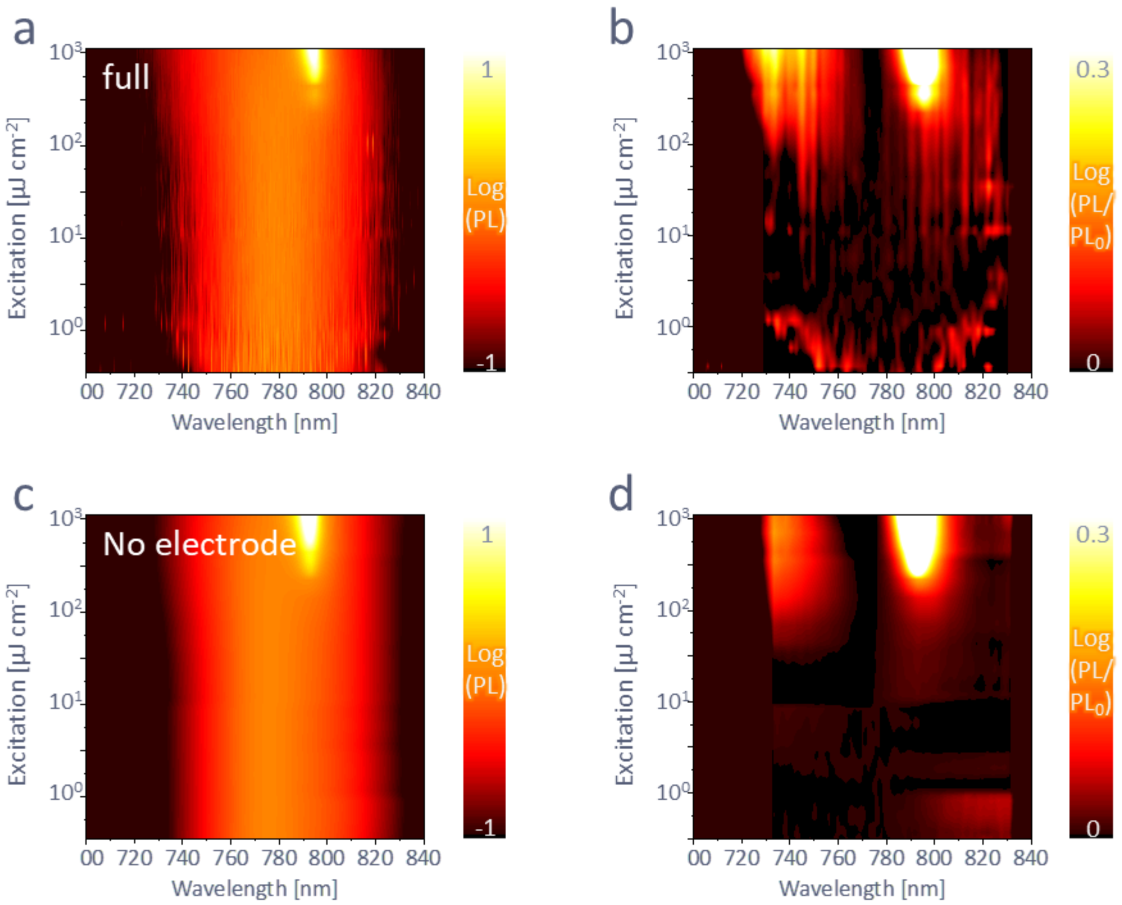

Figure S5. PL spectra (a, c) and PL/PL_0 (b, d) of our full diode structure (a-b) and that without ITO and Ag electrodes (c-d) at various optical excitation intensities (1.3 ns pulse laser, 355 nm, 10 kHz). Each PL is normalized by the value at 775 nm. PL_0 is PL at low excitation. PL/PL_0 more clearly shows the spectral change at short wavelength (near 745 nm) and long wavelength (near 795 nm) at high excitation.

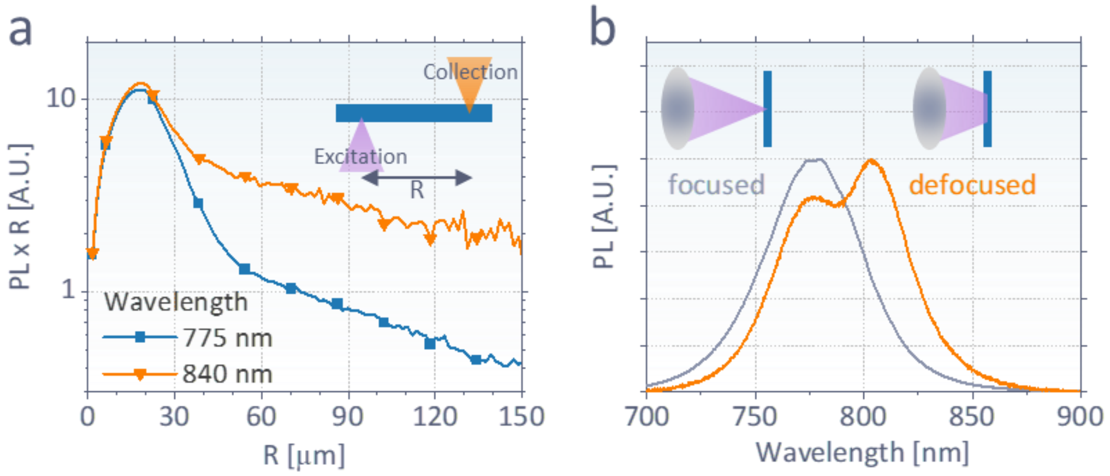

Figure S6. a) Spatially resolved PL by sweeping the lateral distance (*R*) between the excitation and collection objective lenses. Our full device stack without electrodes is illuminated by a pulse laser (5.7 nJ, 355 nm, 10 kHz) using a high NA lens. b) PL spectra at *R* = 0 when the excitation is focused and defocused, respectively.

Figure S6a shows the spatially-resolved PL for the full device stack excluding the metal layer for better signal collection. While reabsorption of perovskite causes faster decay at 775 nm, PL at 840 nm, where perovskite absorption is negligible, shows the broader spread. The decay slope of ~100 cm^{-1} at 840 nm corresponds to the optical loss present for waveguide mode in the device stack, such as scattering and parasitic absorption. Here, when the laser illumination is highly focused in a diffraction limit, no ASE is shown despite the strong excitation (>> 10^3 µJ cm^{-2}), due to the insufficient gain length for ASE.[5g] On the other hand, when the light source is defocused by vertically moving the lens, ASE peak appears despite the diluted excitation density, as shown in Figure S6b. The result shows the limitation of the pixel area, of which minimum area needs to be sufficient to get signal amplification.

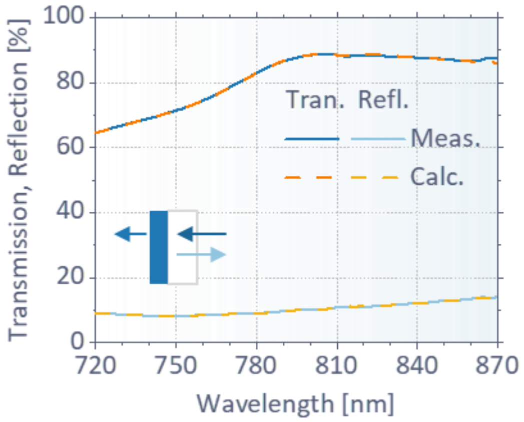

Figure S7. Measured and calculated transmission and reflection of our $Cs_{0.05}FA_{0.95}Pb(Br_{0.1}I_{0.9})_3$ perovskite film. Refractive index of the perovskite shown in Figure 3a is fitted to match the measured and calculated transmission and reflection. Imaginary part of the refractive index (*k*) at long wavelength (> 800 nm), where absorption is too small to be resolved, is fitted using PL spectrum and Boltzmann distribution, in a way described in the previous work. [5g]

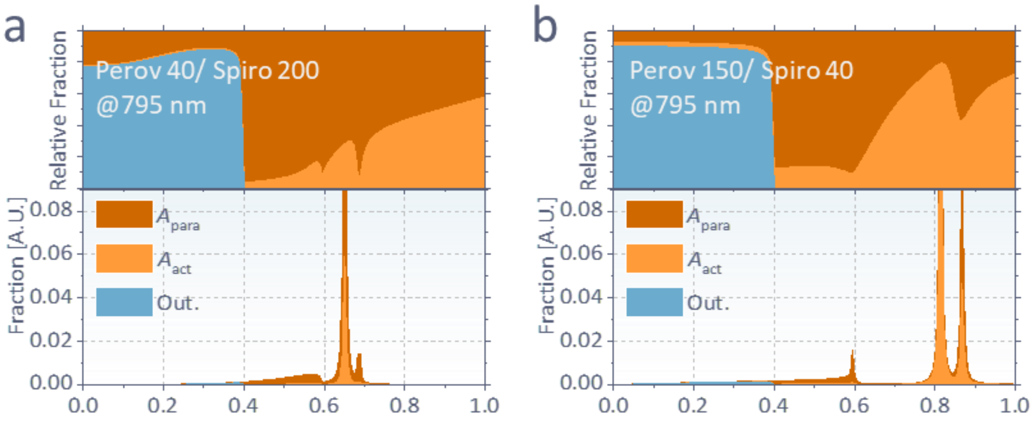

Figure S8. Fraction of optical energy (A_{para}, A_{act}, or outcoupled) as a function of relative radial propagation vector (k_r/k_s) at wavelength of 795 nm, in device structures with different perovskite and spiro-OMeTAD thicknesses.

In addition to Figure 3d-f, Figure S8a and b show the mode fractions at 795 nm, for the structures of perovskite 40 nm/ spiro-OMeTAD 200 nm and perovskite 150 nm/ spiro-OMeTAD 40 nm, respectively. By comparing Figure S8a and Figure 3d, the reduced thickness of perovskite is responsible for the loss of large angle peak (k_r/k_s = 0.84) in Figure 3f. Moreover, in Figure S8a, although thick spiro-OMeTAD layer prevents the loss from Ag absorption, the relative fraction of A_{para} is shown to be large (> A_{act}) even at large k_r/k_s, due to the increased number of bounces in waveguide mode, which cause the increased absorption from ITO side. On the other hand, in Figure S8b having thicker perovskite and thinner spiro-OMeTAD, large angle waveguide mode is well formed and the peaks at large k_r/k_s (>0.8) appear again. However, thin spiro-OMeTAD fails to prevent the Ag absorption and A_{para} is shown to be much larger than that in Figure 3e.

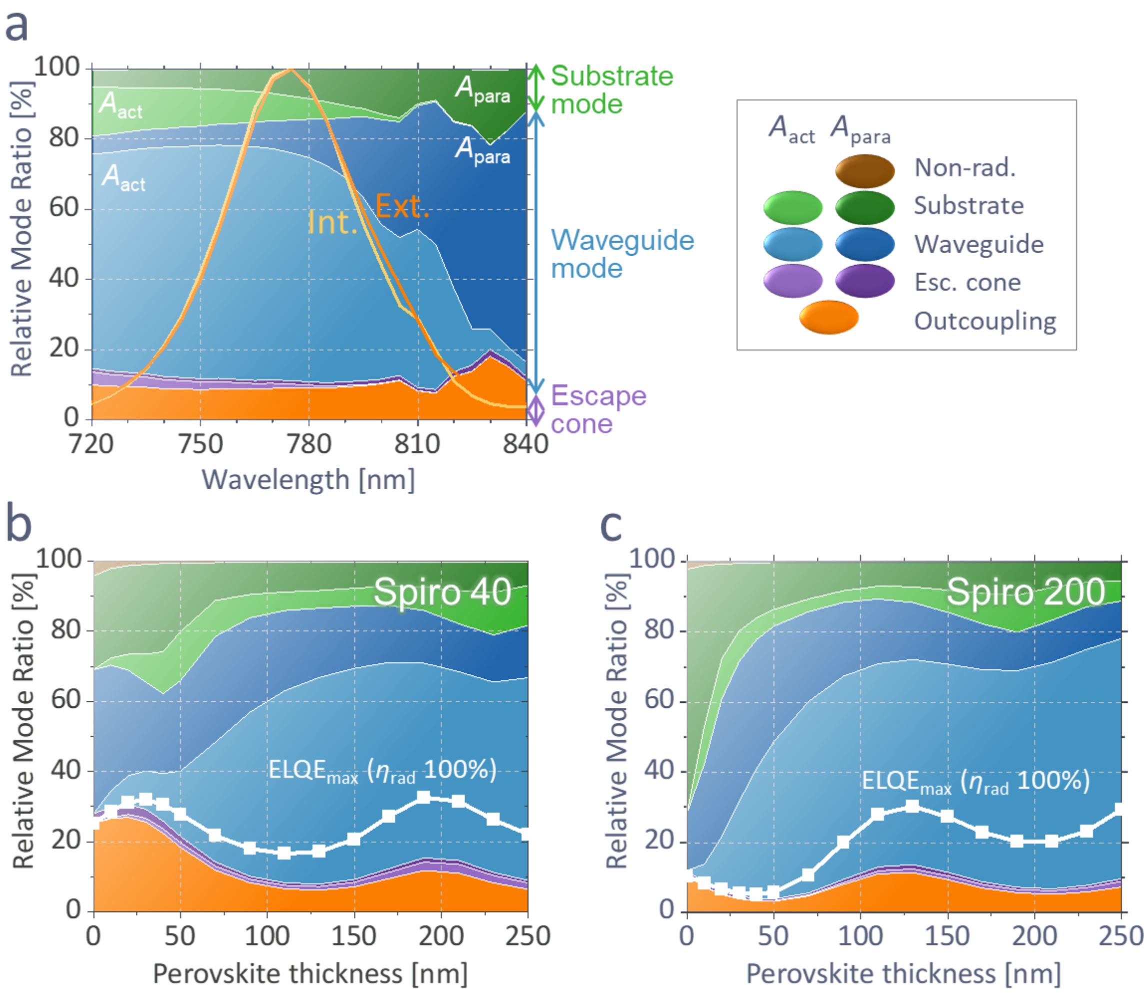

Figure S9. a) Relative fractions of A_{act} and A_{para} in each propagation mode in a perovskite LED at each wavelength. A structure of glass/ ITO 150 nm/ PEIE-modified ZnO 30 nm/ $Cs_{0.05}FA_{0.95}Pb(Br_{0.1}I_{0.9})_3$ perovskite 150 nm/ Li,Co-doped spiro-OMeTAD 200 nm/ Ag is used. Yellow and orange lines indicate the spectra of assumed internal emission and resulted external emission (without photon recycling), respectively. b-c) The integrated mode fractions as a function of perovskite thickness, having 40 nm-thick (b) or 200 nm-thick (c) spiro-OMeTAD. The white line indicates the maximum achievable ELQE with a photon recycling effect when η_{rad} = 100%.

Figure S9 provides more detailed optical information of the device. Photon propagation is divided into escape cone, waveguide mode, substrate mode, and non-radiaitve mode (also called surface plasmon polariton mode). Then each mode is again split into outcoupling (only for escape cone), $\underline{A}_{\mathrm{act}}$, and A_{para}. As shown in Figure S9a, due to larger perovskite absorption coefficient, $\underline{A}_{\mathrm{act}}$ is more dominant at short wavelengths, while A_{para} is relatively more at long

wavelength. In addition to waveguide mode, substrate mode also contributes to A_{act} and causes photon recycling effect, however, it rarely assists ASE as photons in substrate mode typically propagate a long distance (> 1 mm) and the energy is diluted over such long range. Figure S9b-c shows insight for device optimization, to get enhanced ELQE. At each device structure, mode fractions for all conditions of wavelength, dipole orientation, polarization, and dipole position are integrated. With a thin optical spacer (spiro-OMeTAD) of 40 nm, the highest direct outcoupling efficiency is shown at thin perovskite of 30 nm, with an optimal microcavity effect. The optimal thickness is slightly thicker for $ELQE_{max}$, as thicker perovskites bring more benefits from photon recycling effect. Such optimal thicknesses of perovskite shown for a thin optical spacer validate the recent trend of perovskite LEDs mostly having a thin (40~60 nm) perovskite layer. On the other hand, when the spacer is thicker (200 nm), the condition for microcavity effect is changed and optimal thickness is shifted – e.g. 130 nm in Figure S9c. As thicker perovskite dilutes the optical resonance causes more reabsorption, even at 130 nm, direct outcoupling efficiency is shown to be only half of the highest value of Figure S9c. However, when photon recycling is taken into account, the thicker perovskite can get more benefits through larger A_{act}, hence $ELQE_{max}$ at 130 nm in Figure S9c is shown to be just similar as the maximum values in Figure S9b. Although thick perovskite and thick spiro-OMeTAD layers are used to maximize the waveguide mode and get ASE property in our work, the results in Figure S9 imply an opportunity for those structures to work also as efficient LEDs.
